# Maturation of the Intestinal Epithelial Barrier in Neonatal Rats Coincides with Decreased FcRn Expression, Replacement of Vacuolated Enterocytes and Changed Blimp-1 Expression

**DOI:** 10.1371/journal.pone.0164775

**Published:** 2016-10-13

**Authors:** Ester Arévalo Sureda, Björn Weström, Stefan G. Pierzynowski, Olena Prykhodko

**Affiliations:** 1 Department of Biology, Lund University, Lund, Sweden; 2 Innovation Centre STB, Tczew, Poland; National Cancer Institute, UNITED STATES

## Abstract

**Background:**

The intestinal barrier is immature in newborn mammals allowing for transfer of bioactive macromolecules, *e*.*g*. protecting antibodies, from mother’s milk to the blood circulation and in neonatal rodents lasts until weaning. This passage involves the neonatal-Fc-receptor (FcRn) binding IgG in the proximal and highly endocytic vacuolated enterocytes in the distal immature small intestine (SI). Recent studies have suggested an involvement of the transcription factor B-lymphocyte-induced maturation-protein-1 (Blimp-1) in the regulation of SI maturation in mice. Hence, the objective of the present study was to monitor the development of the intestinal barrier function, in relation to Blimp-1 expression during both natural and precociously induced intestinal maturation in rats.

**Results:**

During the suckling period IgG plasma levels increased, while after gut closure it temporarily decreased. This corresponded to a high expression of FcRn in the proximal SI epithelium and the presence of vacuolated enterocytes in the distal SI. The immature foetal-type epithelium was replaced after weaning or induced precocious maturation, by an adult-type epithelium with FcRn^neg^ cells in the proximal and by non-vacuolated enterocytes in the distal SI. In parallel to this epithelial shift, Blimp-1 expression decreased in the distal SI.

**Conclusion:**

The switch from foetal- to adult-type epithelium, with decreased proximal expression of FcRn and distal replacement of vacuolated enterocytes, was concurrent in the two SI regions and could be used for monitoring SI maturation in the rat. The changes in expression of Blimp-1 in the distal SI epithelium followed the maturation pattern.

## 1. Introduction

The stage of gastrointestinal maturation at birth varies among different mammalian species and rodents are altricial species born immature with final structural and functional organ development taking place postnatally [1, 2]. Thus, the neonatal rat is suitable as a model for the study of the barrier function and immune system in the gut and their possible connections during maturation [3, 4].

In neonatal rats the immature gastrointestinal tract is adapted to the digestion of milk but permeable to essential milk-borne bioactive macromolecules like antibodies, growth factors and cytokines. Macromolecules are transferred through the SI epithelium via transcellular endocytosis from the lumen into the blood circulation [5]. Receptor-mediated transcytosis of milk-borne immunoglobulin G (IgG) for acquisition of passive maternal immunity takes place under protection from degradation by binding to the neonatal-Fc-receptor (FcRn) [6–8]. The presence of FcRn in the epithelium of proximal SI has been shown by measurement of the receptor’s binding capacity for IgG [8, 9] and mRNA quantification [10], but protein expression and localisation of FcRn in the SI has not yet been monitored during postnatal development. The presence of enterocytes with a large supranuclear digestive vacuole is an evident morphological feature of the distal SI in suckling rats [11] and non-selective endocytosis might also contribute to the trans-epithelial passage of macromolecules affecting the SI barrier properties [1].

During the third week of life in the rat, when the transition from a milk-based to a solid food diet commences (the weaning period), gut growth and maturation accelerate involving both functional and structural changes in the SI epithelium [12]. During this process the intestinal barrier properties increase resulting in a markedly reduced permeability to macromolecules known as gut closure. This is demonstrated by the ceased uptake of proteins, like bovine serum albumin and IgG, as well as non-protein macromolecular markers, *i*.*e*. FITC-dextrans [13–15]. To study these ontogenic changes, gut maturation can be induced precociously before natural weaning and we have shown that this is possible in a dose-dependent and irreversible manner by feeding suckling rats with a lectin from red kidney beans (PHA) or pancreatic (or pancreatic-like) proteases [14, 15].

The transcription factor B lymphocyte-induced maturation-protein-1 (Blimp-1) was recently shown expressed in the intestinal epithelium of mice during the foetal and neonatal periods, while at weaning the expression of Blimp-1 abruptly decreased [16, 17]. Blimp-1 was suggested to play a key-role in repressing the intestinal epithelial maturation until weaning. However, the expression of Blimp-1 and its role during postnatal maturation of the SI epithelium has not yet been studied in other species, such as rats.

The aim of the present study was to follow the maturation process in the SI during natural and precociously induced maturation in the rat, by monitoring the intestinal barrier properties as well as changes in the epithelial expression of FcRn and the presence of vacuolated cells in relation to the transcriptional repressor Blimp-1.

## 2. Material and Methods

### 2.1. Animals

The Malmö-Lund’s Ethical Committee on Animal Experiments approved the study (Permit number: M228–11) designed according to the European Parliament and Council Directive (2010/63/EU) and the Swedish Animal Wellfare Act (SFS 1988:539). The study was performed on rats (*Rattus Norvegicus*) of the Sprague-Dawley strain (Mol:SPRD Han; Taconic M & B A/S, Denmark) which were bred in the departmental animal facility using an open-cage system under specific pathogen-free conditions (20 ± 1°C, 50 ± 10% RH and a 12:12 hours light-dark cycle). The rats were kept in polycarbonate cages (Macrolon III) on a chopped aspen wood bedding (Beekay B & K Universal AB, Sweden) enriched with paper-nesting material and with free access to tap water and a laboratory rodent chow (RM1, SDS, England) from a lid feed hopper. The dams were individually housed from one week before parturition, the date of birth of the rat pups was designated as day 0 and the litter size was restricted to 10–12 pups within 1–3 days. The pups were kept with their dam until postnatal day 21, after which the dam was separated from her litter.

### 2.2. Experimental Design

The experiments were performed in a split-litter manner where the rat pups were randomly divided into different experimental groups within the litters. Natural development of the SI was studied in five age groups of litter-mates from 3 litters: suckling rats, 7 days (n = 6), 14 days (n = 6), and 21 days old (n = 6), which coincided with the day of separation from their dam, and two post-weaning age groups at 28 days (n = 7) and 35 days old (n = 6). In addition, 42, 49 and 56 days old rats (n = 6–10) were included for blood plasma collection.

Induced precocious maturation was studied in 17 days-old litter-mates, from 2 litters and included two treatment groups and one control group. Fourteen day-old suckling rats were gavaged once a day for three days (14–16 days of age) with either a proteinase from *Aspergillius melleus* (type XXIII, Sigma-Aldrich Co, USA) [15] at a dose of 0.4 mg/g b.wt. (n = 6) or the purified lectin PHA from red kidney beans (*Phaseolus vulgaris*) [14, 18] at a dose of 0.05 mg/g b.wt. (n = 6) [14, 18] dissolved in water while the control group only received water (n = 6). During the study period a 7 cm wall extender was inserted into the cage to prevent pups from reaching the solid food in the lid hopper.

### 2.3. Material Collection

At the day of euthanasia the rats were anesthetized with a subcutaneous injection of a mixture of ketamine (Ketalar^®^, Pfizer, USA; 0.17 mg/g b.wt.) and azaperone (Stresnil^®^, Janssen Pharmaceutica, Belgium; 0.03 mg/g b.wt.) prior to sample collection. To ensuring deep anesthesia the eyelid and withdrawal reflex were checked before opening the thorax. Then, 1 ml of blood was collected via cardiac-puncture into a syringe containing a mixture of 1.5 mg EDTA and 20 KIU of a protease inhibitor (Trasylol^®^, Bayer HealthCare AG, Germany). The blood was centrifuged at 3000 x *g* for 15 min at + 4°C, the plasma was harvested and stored at—20°C until analysis. Next, the SI was dissected from the pylorus to the ileo-cecal junction and divided into a proximal and a distal half. The luminal content was flushed out with ice cold 0.9% NaCl. Intestinal samples, approximately 1 cm-long, were taken from the middle of each region, fixed in 10% neutral buffered formalin for 24 hours at room temperature and then kept in 70% ethanol until paraffin embedding, according to the standard procedure.

### 2.4. Histology and Immunohistochemistry

The intestinal samples were sliced into 5 μm-thick sections, deparaffinized and stained with haematoxylin and eosin (H & E) according to standard procedures. Prior to each immunohistostaining procedure the endogenous peroxidase activity was blocked by incubation with Peroxidized 1 reagent and with Background Sniper to reduce the background (MACH 1 and 4 Universal; Biocare Medical, Llc., USA). The sections were then incubated with the primary antibodies: polyclonal rabbit anti-rat-FcRn (M-255; Santa Cruz Biotechnology, Inc., USA; diluted 1:600) or polyclonal rabbit anti-Blimp-1 (PA5-20310, Invitrogen, ThermoFisher Scientific Inc; diluted 1:40000), in 0.02 M PBS containing 1% bovine serum albumin (BSA), overnight at + 4°C. The next day, staining using the HRP-Polymer Detection kit (MACH 1 for FcRn, and MACH 4 for Blimp-1, Universal Detection kits; Biocare Medical, Llc., USA) was performed according to the manufacturer’ specifications and using 3,3-diaminobenzidine as a substrate. Finally, the sections were counter-stained with haematoxylin, dehydrated and mounted under a cover slip using DPX medium (BDH chemicals Ltd., England). Sections in which the primary antibody had been replaced by only PBS + 1% BSA were included as a control for unspecific binding of the HRP-Polymer detection kit to the tissue. In addition, prior to Blimp-1 staining procedure, slides were subjected to antigen retrieval by microwaving 2 × 8 min at 650 W in TRIS-EDTA buffer (0.01 M, pH 9). The specificity of the primary anti-Blimp-1 antibody was verified by pre-incubating it during 30 min at RT with the blocking peptide that corresponds to 14 amino acids near the carboxy terminus (ratio 1:5 antibody:peptide, PEP-0430, Invitrogen, ThermoFisher Scientific Inc.) and followed the same immunostaining procedure (S1 Fig).

### 2.5. mRNA Expression by RT-qPCR

Reverse transcription of RNA followed by quantitative polymerase chain reaction (RT-qPCR) was performed for Blimp-1 (Prdm1) and FcRn (Fcgrt) mRNA. Total RNA from proximal and distal portions of SI was extracted using the RNeasy® Mini Kit (Qiagen) according to the manufacturer's instructions. Genomic DNA was eliminated during RNA extraction by using RNase-free DNase set (Qiagen) according to instructions. Total RNA concentration was determined by using Qubit® RNA HS assay kit (Life Technologies) in a Qubit® 2.0 fluorometer (Invitrogen) and 50–200 ng of total RNA was used for reverse transcription (RT) per reaction. The RT reactions were performed with the RevertAid First Strand cDNA Synthesis Kit (Thermo Scientific™) according to the manufacturer's protocol. The amount of cDNA was measured with Qubit® ssDNA assay kit (Life Technologies).

RT-qPCR was performed using a C1000 Touch Thermal Cycler (BioRad) on 5–10 ng (2 μl of a 20 μl RT reaction) of first strand cDNA using SsoAdvanced™ Universal SYBR® Green Supermix (BioRad laboratories, USA) in triplicates, according to the manufacturer's instructions. The primers used were predesigned on the rat sequence by the manufacturer (KiCqStart^®^ SYBR^®^ Green Primers, Sigma-Aldrich) and ribosomal protein L13 (Rpl13a) was used as a housekeeping gene. The sequence of the primers used were as follows: Prdm1 F: ATTTTTGGCGGATCTATTCC / R: AGGGATAGGCTTAATAGTGTAG; Fcgrt F: AAATAAATGGGACCTTCACAC / R: ACCAACGATATCTGTCTCC; Rpl13a F: AGTTAAAGTATCTGGCCTTTC / R: CTCTTTTGGTCTTGTGCG. Amplification of the PCR products was preformed as follows: initial denaturing at 95°C, 3 min, followed by 40 cycles (denaturing at 95°C, 15 sec, annealing at 58°C, 30 sec and a plate read). A melting curve for each primer was included at the end of the program from 65°C to 95°C, with an increment of 0.5°C for 5 sec and plate read. Melting curve analysis of PCR products indicated single products for each primer pair used.

### 2.6. Determination of Plasma IgG

Plasma IgG levels were quantified by single radial immunodiffusion [19] using rabbit anti-rat-IgG (DAKO A/S, Denmark) as the precipitating antibody. Purified rat IgG (Miles Laboratories Inc.; USA) was used as the standard, and sample concentrations were interpolated from the standard curve that was generated.

### 2.7. Measurements, Calculations and Statistics

Microscopic examination was performed using an Olympus PROVIS microscope connected to an Olympus DP50 camera (Olympus, Japan), and the images were evaluated by morphometry using the Image*J* software (National Institutes of Health, Bethesda, MD, USA). The proportion, in percent, of adult-type enterocytes in the villi epithelium was estimated by measuring the length of FcRn^neg^ cells in the proximal SI and the length of non-vacuolated enterocytes in the distal SI, in relation to the total villous length.

One-way ANOVA with multiple comparisons and Tukey’s post-hoc test was performed for analysis of plasma IgG levels during natural development, while Dunnett’s post-hoc test was used for analysis of plasma IgG levels in precociously-induced maturation groups and analysis of the proportion of adult-type epithelium between groups during natural development and in induced maturation groups. The percentage of adult-type epithelium in treatment groups was also plotted for correlation with R^2^ and Pearson and Spearman test calculated. All statistics were performed using GraphPad Prism version 7 for Mac (GraphPad Software, San Diego, California, USA, www.graphpad.com). Significance was considered when p < 0.05 (*), p < 0.01 (**), p < 0.001 (***), p < 0.0001 (****), or non-significant (ns).

## 3. Results

### 3.1. Morphological Changes during Development

Microscopic examination of the SI samples revealed characteristic morphological changes with age (S2 Fig), such as the transition from finger-shaped villi in suckling rats (7d and 14d) to more tongue-shaped villi in weaned rats (28d and 35d). Also evident crypt formation from 14 days of age and an increase in goblet cells, distinctive for their spherical shape and non-stained cytoplasm, in the villi epithelium was found. Infiltration of immune cells into the *lamina propria* and signs of epithelial damage in the villi tips could be seen in rats during and after weaning (21d and 28d).

**Fig 1:**
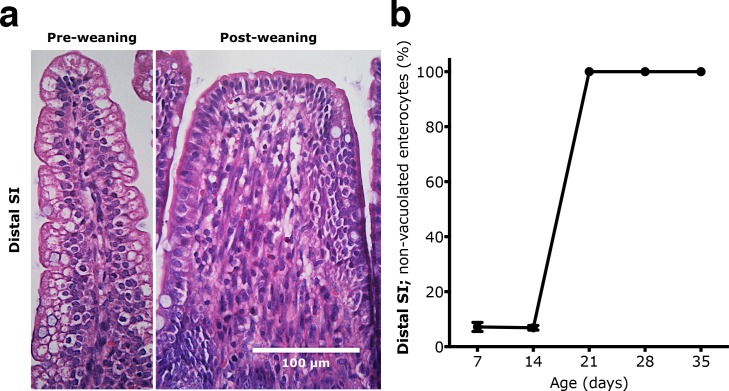
Replacement of vacuolated foetal-type epithelium by non-vacuolated adult-type epithelium in the distal part of the SI during postnatal development in rats. (**a)** Representative H & E stained sections (magnification 400x) showing the structural changes in the distal SI between the suckling (7d) and the post-weaning periods (28d). (**b)** Appearance of adult-type epithelium with non-vacuolated cells (in % of total villous length) in 7, 14, 21, 28 and 35 days old rats (mean ± SD, n = 6–7).

A major difference was obvious along the proximal-to-distal axis of the SI, since in the distal part vacuolated enterocytes were the predominant epithelial cell-type in suckling rats (7d and 14d). However, from day 21 these foetal-type cells had been replaced by non-vacuolated enterocytes—adult-type cells—from the villous base and the morphological differences between the proximal and distal SI were reduced (Fig 1). The proportion of non-vacuolated, adult-type epithelial cells along the villi, increased from 6.8 ± 0.8% to 100% between 14 and 21 days of age.

**Fig 2:**
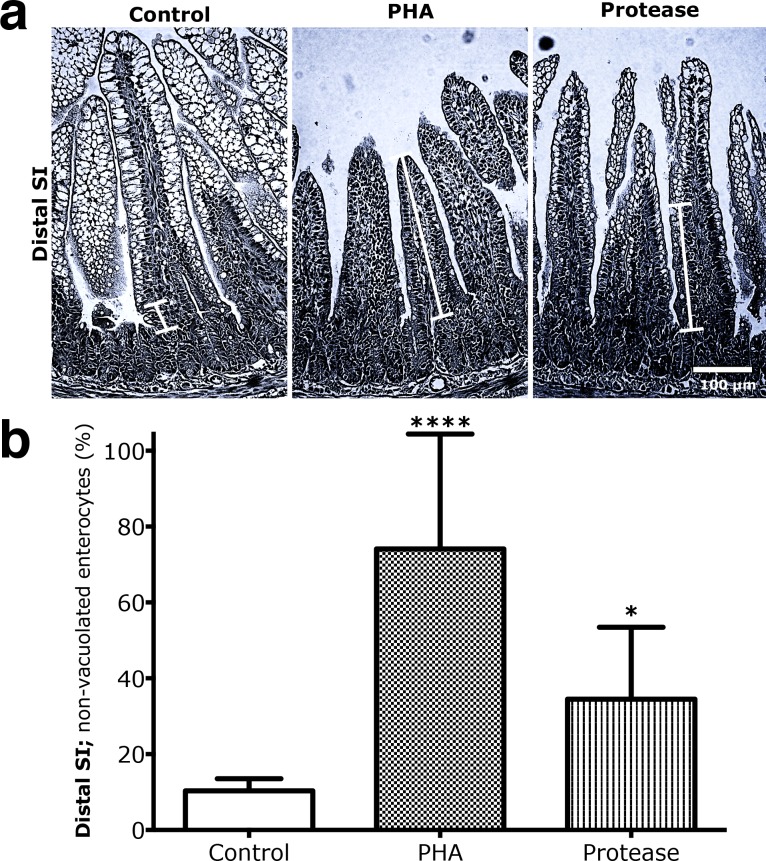
Increased replacement of vacuolated foetal-type epithelium by non-vacuolated adult-type epithelium in the distal part of the SI in rats due to precociously-induced maturation. **(a)** H & E stained sections (magnification 200x) from the distal SI in 17 days old rats gavage treated with PHA or protease for 3 days, to induce precocious gut maturation, compared to control rats gavaged water. (**b)** The proportion of adult-type non-vacuolated enterocytes appearing in the distal SI epithelium (in % of total villous length) in 17 days old rats treated with PHA or protease compared to control rats (mean ± SD, n = 6; * p < 0.05; **** p < 0.0001).

Precociously induced maturation in 17 days old rats, resulted in a significantly increased proportion of the non-vacuolated enterocytes in the distal SI, from 10.3 ± 2.8% in the control group to 74.1 ± 27.1% in the PHA treated group (**** p < 0.0001) and 34.5 ± 17.3% for the protease treated group (* p < 0.05), with vacuolated enterocytes only remaining at the villi tips (Fig 2). Thus, during both natural and precociously induced development, a gradual replacement of the foetal-type vacuolated cells by adult-type enterocytes, progressing from the base to the tip of the villous, was seen in the distal SI.

### 3.2. Expression of FcRn during Development

**Fig 3:**
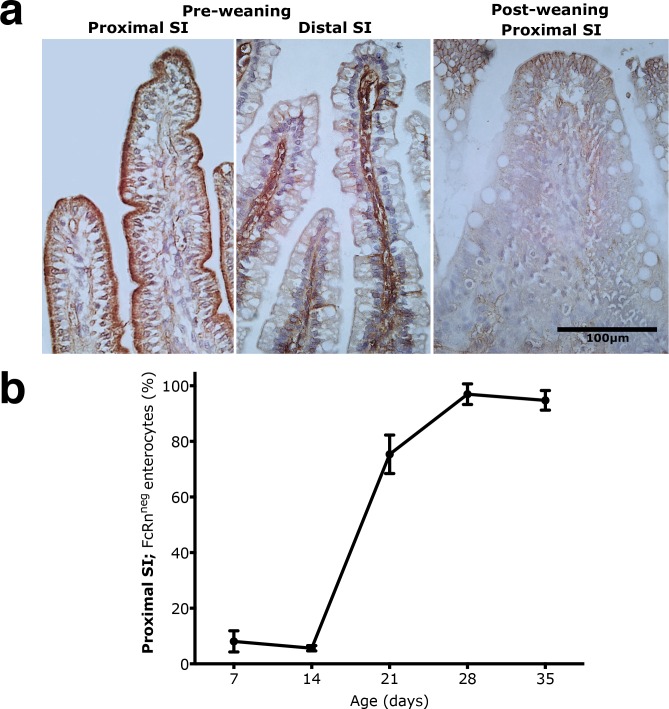
Replacement of FcRn^pos^ foetal-type epithelium by FcRn^neg^ adult-type epithelium in the proximal part of the SI during postnatal development in rats. **(a**) Immunohistochemistry of FcRn shown in representative histological sections (magnification 400x) in the proximal SI from the suckling (7d) to the post-weaning periods (28d). For comparison, note the lack of staining for FcRn in the epithelium, while staining seen in the *lamina propria* in the distal SI of suckling 7 days old rats. (**b)** Appearance of adult-type epithelium with FcRn^neg^ enterocytes (in % of total villous length) in the proximal SI of 7, 14, 21, 28 and 35 days old rats (mean ± SD, n = 6–7).

Immunohistochemical staining showed a strong expression of FcRn in the apical portion of the epithelial cells in the proximal SI of suckling rats (7d and 14d), with increasing intensity along the crypt-villus axis (Fig 3A and S3 Fig). In 21 days old pups, FcRn expression was reduced and restricted to the upper half of villi, which became even more evident in the post-weaning rats (28d and 35d). The replacement of FcRn^pos^ cells by FcRn^neg^ cells in the proximal SI epithelium was 75.3 ± 6.9% by the third week (21d), and 96.9 ± 3.7% in 28 days old pups (Fig 3B).

In the distal SI epithelial FcRn expression appeared lower than in the proximal region, but individual disperse cells in the upper half of the villi were highly positive (S3 Fig). The 21 days old rats showed decreased staining of FcRn, being mainly localised apically in epithelial cells at the villi tips, and in the post-weaning groups (28d and 35d), these cells were further limited, although some disperse epithelial cells still showed FcRn stained cytoplasm.

**Fig 4:**
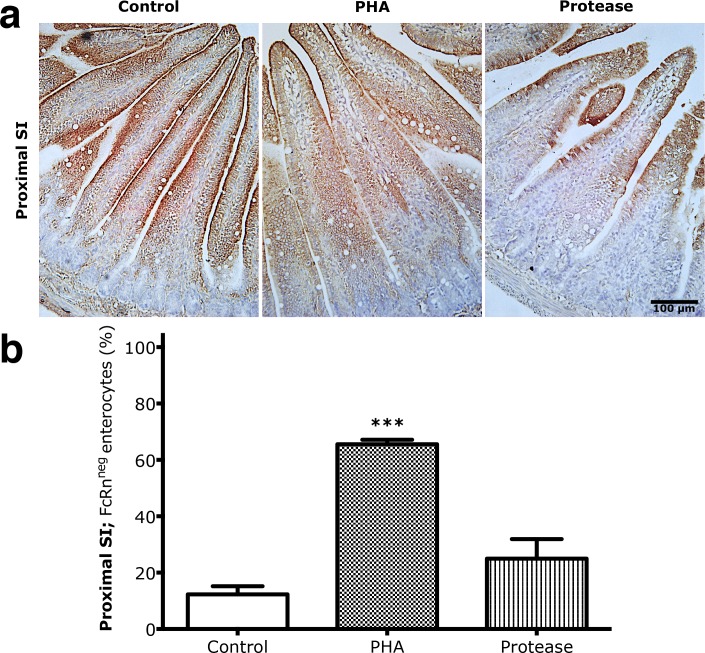
Increased replacement of FcRn^pos^ foetal-type epithelium by FcRn^neg^ adult-type epithelium in the proximal parts of the SI after precociously induced maturation in rats. (**a)** Immunohistochemistry of FcRn in representative histological sections (magnification 200x) from the proximal SI in 17 days old rats gavaged with PHA or protease for 3 days to induce precocious maturation, compared to control rats. (**b)** The proportion of adult-type FcRn^neg^ enterocytes in the proximal SI epithelium (in % of total villous length) in 17 days old rats treated with PHA or protease, compared to control rats (mean ± SD, n = 6; *** p < 0.001).

**Fig 5:**
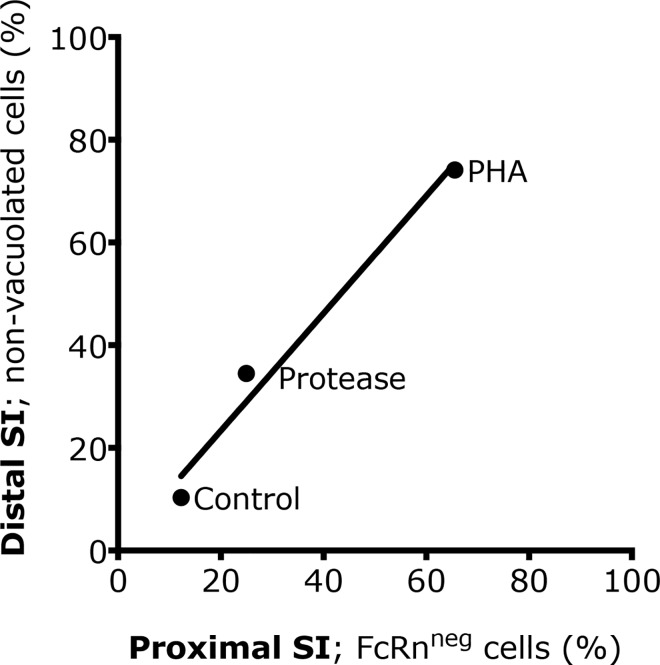
Correlation between the maturational appearance of adult-type epithelium in the proximal and the distal SI. Appearance of FcRn^neg^ cells in the proximal SI and non-vacuolated cells in the distal SI in 17 days old rats treated with PHA or protease for 3 days, to induce precocious gut maturation, compared to control rats.

After induced precocious maturation, due to PHA or protease exposure, the expression of FcRn was reduced, remaining apically in epithelial cells at the upper part of the villi in the proximal SI, while controls expressed FcRn along the villi epithelium (Fig 4). Thus, the presence of FcRn^neg^ cells in the PHA treatment group (72.75 ± 8.7%) was significantly increased (*** p < 0.001) compared to controls (12.3 ± 2.5%) while protease treatment had no significant effect (Fig 4). A high correlation (Pearson coefficient, r, and R^2^ = 0.98) was found between the degree of replacement of the foetal-type by adult-type SI epithelium in the proximal and distal regions (Fig 5), *i*.*e*., FcRn^neg^ enterocytes in the proximal and non-vacuolated cells in the distal SI, indicating that the effects in the two studied regions occur in parallel during both natural and induced maturation.

In addition to the epithelial FcRn expression, staining was also found in the villi *lamina propria*, including immune cells, being weak in the proximal SI while stronger in the distal SI in the suckling rats (7d and 14d) (Fig 5). In older rats, FcRn appeared in the *lamina propria* with somewhat stronger intensity in the proximal SI and was still detected in the distal SI in 21 days old pups, while in the post-weaning groups (28d and 35d) staining had decreased. After induced maturation, in both the protease and PHA treated groups, an increased number of FcRn stained cells appeared within the *lamina propria* in the distal SI, compared to the control group (S4 Fig).

**Fig 6:**
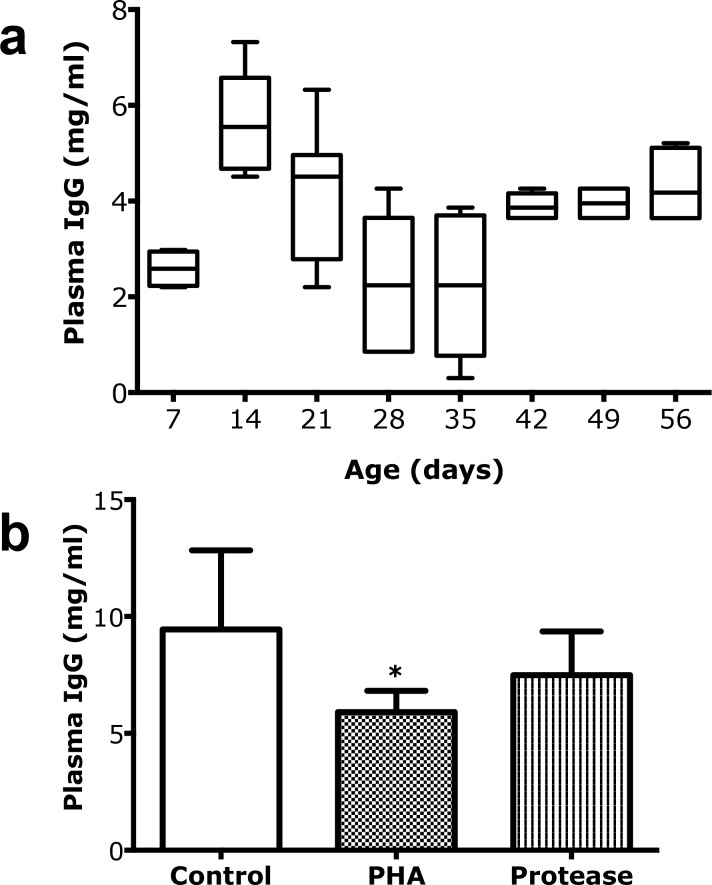
Expression of mRNA for Fcgrt (FcRn) and Prdm1 (Blimp-1) in the SI of developing rats. Relative mRNA levels of Fcgrt **(a, c)** and Prdm1 **(b, d)** in the proximal and distal SI in 7, 14, 21, 28 and 35 days old rats (n = 4–6) during postnatal development (a, b) and in 17 days old rats treated with PHA or protease at 14–16 days of age (n = 3–4) to induce precocious gut maturation, compared to control rats (c, d). (mean ± SD, a–b and * p < 0.05; *** p < 0.001).

The RT-qPCR analysis showed a significant decrease of mRNA for FcRn (Fcgrt) expression with age/induced maturation in the proximal SI that support the results obtained with immunohistochemistry (Fig 6A and 6C).

### 3.3. Plasma Levels of IgG

**Fig 7:**
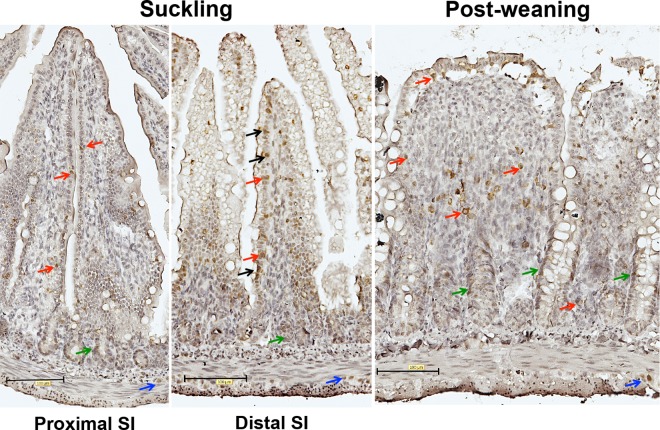
Plasma IgG as an accumulative marker for intestinal macromolecular permeability. **(a)** Changes in plasma IgG levels (mg/ml) during postnatal development in suckling rats, 7, 14, and 21 days old and post-weaning rats, 28, 35, 42, 49 and 56 days old (mean ± SD, 10–90% percentile, (boxes), n = 6–10). **(b)** Decreased plasma IgG levels (mean ± SD, n = 6) in 17 days old rats treated with PHA (* p < 0.05) or protease (non-significant) during 3 days to induce precocious maturation, compared to control rats.

The level of IgG in blood plasma increased in suckling rats reaching a peak in the 14 days old pups and then decreasing to a minimum after weaning (28d and 35d) (Fig 7A). The change in plasma IgG levels showed an inverse correlation (r = -0.65) with the appearance of adult-type FcRn^neg^ cells in the proximal SI during this age period (S4 Fig). In older rats, the plasma IgG levels started to increase again until reaching a plateau of around 4 mg/ml after 42 days of life. After induced maturation in the rats treated with PHA or protease, the plasma IgG levels was lower, for PHA significatly, compared to that of the controls (Fig 7B).

### 3.4. Expression of Blimp-1 during Development

**Fig 8:**
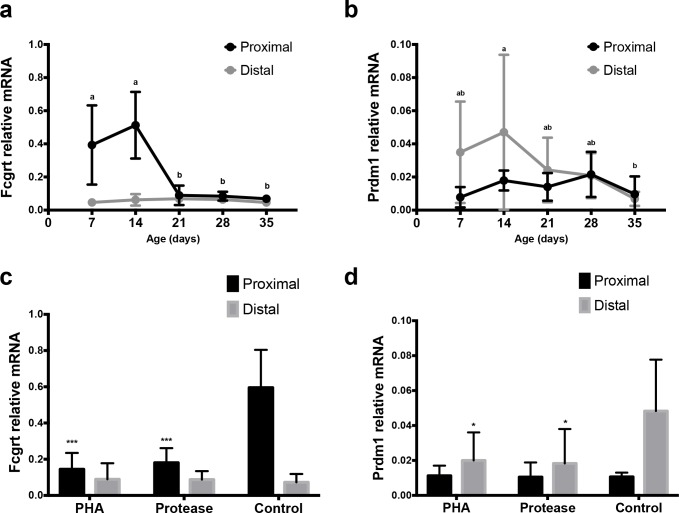
Expression of Blimp-1 in the small intestine during suckling and post-weaning periods. Immunohistochemical staining of Blimp-1 in representative histological sections (magnification 200x) from the proximal and distal SI in suckling (14d) and post-weaning (28d) rats. **Note:** Nuclear immunorreactivity of Blimp-1 antibodies in epithelial cells indicated by black arrows; intraepithelial and lamina propria cells–red arrows; crypt cells–green arrows and cells in muscle layer are indicated by blue arrows.

Epithelial cells along the villi were positively stained with anti Blimp-1 antibodies in the distal part of SI of suckling rats, especially at age of 14 days old, with some variation between villi and individuals (Fig 8). The nuclear staining disappeared from the villi epithelium in the post-weaning age groups. On the other hand, the immunostaining of Blimp-1 was not evident in the epithelial cells of the villi in the proximal SI in all ages.

Additionally, immunostaining of Blimp-1 was observed in the crypt-region cells in both the proximal and the distal parts of the SI in all ages. Blimp-1 immunostaining was found in epithelium-associated cells, in the lamina propia, in the submucosa and in cells between the muscular layers.

The RT-qPCR analysis of Blimp-1 (Prdm 1) mRNA showed decreased expression in the distal part of the SI in the post-weaning age groups, while no obvious changes were observed in the proximal SI during development (Fig 6B). Similarly, PHA- and protease-induced precocious maturation resulted in decreased mRNA Blimp-1 expression in the distal part of the SI (Fig 6D).

## 4. Discussion

### 4.1. Intestinal Barrier Function and Plasma IgG Levels

In the present study, intestinal barrier function was monitored by measuring the plasma levels of IgG, as an accumulative marker for macromolecular passage from mothers milk to the neonate’s blood circulation [20]. Thus, during the first three weeks after birth, continuously increasing plasma levels of IgG were seen reflecting the immature SI epithelium permeable to macromolecules, like IgG, during the suckling period. After this, at weaning, the plasma IgG levels decreased markedly due to the decreased permeability at intestinal closure [10, 13]. From postnatal day 35 the plasma IgG levels restarted to increase, which can be attributed to the activation of the adaptive immunity [21], with an increased antibody (IgG) production due to the exposure to new dietary and microbial antigens after weaning. The high permeability of the SI during the suckling period was reflected in the presence of the immature SI epithelium, evidenced by the histological and FcRn immunohistochemistry and mRNA analysis.

### 4.2. Neonatal-Fc-Receptor Expression

It is well known that FcRn mediates the binding and transfer of IgG across the SI during the suckling period in rats [11]. Our results, obtained by monitoring the FcRn protein expression in the epithelial tissue by immunohistochemistry, are in line with this as well as with the FcRn mRNA quantification in this study and previously published data [10]. Thus, FcRn was expressed with highest intensity in the proximal SI during the suckling period whereas the expression markedly decreased at weaning. Generally, FcRn was localised in the apical part of the foetal-type epithelial cells with a gradual increase in expression towards the villi tips and with no expression in the crypts. After weaning, in the adult-type epithelium, FcRn was expressed only in a few cells at the villi tips, with a dotted staining in the cytoplasm, indicating a vesicular deposition. This low expression of FcRn in the SI epithelium after weaning might indicate a second role of this receptor contributing to the sampling of luminal antigens and transporting IgG–antigen complexes to the underlying *lamina propria* with immune cells [22].

### 4.3. Presence of Vacuolated Enterocytes

During the suckling period vacuolated enterocytes are the predominant epithelial cell-type in the distal portion of the SI [11]. These foetal-type cells have high endocytic activity and form a large supranuclear vacuole, occupying most of the cytoplasm and have an intracellular digestive function. These distal absorptive cells might, even if they are digestive, contribute to the uptake and transfer of intact macromolecules like maternal IgG, however, in a less specific manner than in the proximal SI, expressing the IgG selective receptor FcRn [23]. The foetal-type epithelium, with vacuolated enterocytes, was totally replaced by the adult-type non-vacuolated epithelium in the distal SI after weaning.

### 4.4. Natural and Induced Maturation

Gavage of PHA and protease for three days accelerated the intestinal maturation, resulting in a precocious maturational status in 17 day old rat pups similar to that observed in the rats after natural weaning (21–28 days old). Thus, the treated groups showed decreased plasma IgG levels compared to controls indicating increased barrier properties, as well as changed epithelial phenotypes in both SI regions, i.e. decreased proximal FcRn expression and disappearance of the vacuolated enterocytes in the distal region. However, the treatments differed in magnitude, since PHA gavage resulted in a total switch to the adult-type epithelium in both parts of the SI, while the effects of the treatment with protease, given in a suboptimal dose, were only about 20–40% of those caused by PHA [15]. Thus, the degree of maturation, naturally or precociously induced, could be followed by the appearance of FcRn^neg^ epithelial cells in the proximal SI and/or non-vacuolated enterocytes in the distal SI. In fact, a strong correlation between the epithelial replacement in the proximal and distal parts of the SI was found and these changes could be used independently or in combination as markers to monitor the developmental maturation of the SI in the young rat.

### 4.5. Expression of Blimp-1

Two recent studies on the SI expression of the transcription factor Blimp-1 in mice suggested its possible role as a regulating-element in gut maturation [16, 17], since young mice showed a cease in Blimp-1 expression in the SI epithelium at weaning correlating with the transition to adult-type epithelium [24]. In the present study done in rats, we also observed expression of Blimp-1 in the nuclei of some distal SI enterocytes in the suckling age groups, which was no longer found in the post-weaning age groups and is in line with the findings in mice [16, 17]. In contrast to the distal part, the proximal SI enterocytes had no evident expression of Blimp-1 and, in fact, data on Blimp-1 in the proximal SI epithelium has not been previously published to our knowledge.

Moreover, we observed an additional localisation of Blimp-1 in the SI, where some cells in the crypt region were found positive, especially in the proximal region in the post-weaning groups. Furthermore, single intraepithelial cells along the villi and cells in the *lamina propria* and submucosa presented clear expression Blimp-1 in their cytoplasm, presumed to be immune cells [25]. We could also observe expressing Blimp-1 cells between the muscle layers, possibly belonging to the myenteric plexus. The expression of Blimp-1 at all rat ages included in the study was confirmed by mRNA analysis. Taken together this suggests that Blimp-1 may function as nuclear transcription repressor in the distal SI enterocytes during the suckling period, presumably delaying SI epithelial maturation, as it was suggested in mice [17]. However, further studies for the characterisation of the cell types expressing Blimp-1 in the SI in rats would be of interest and perhaps Blimp-1 knockout models would be needed to further investigate the role of Blimp-1 in intestinal epithelial maturation.

### 4.6. Connection between the Barrier Function and the Immune Systems in the Gut

In addition to the intestinal barrier, also the immune system of the gut at birth is immature and functionally naive due to the low antigenic stimulation *in utero* [26]. The maternal passive immunity obtained by absorption of milk-borne IgG during the suckling period will provide a temporary protection, but the immune system will also be activated by exposure to the manifold of environmental, dietary and microbial, antigens [27]. The increase in plasma IgG levels evident after weaning, verified the activation of the immune system. The low expression of FcRn in some epithelial cells at the villi tips as well as in the *lamina propria*, endothelial and possibly immune cells, remaining in post-weaning rats (28 and 35 days old) can be related to FcRn’s functions in retrieval of luminal antigens, and the uptake of IgG-antigen complexes for antigen presentation. Thus, FcRn may have a dual role not only in the intestinal transfer of passive immunity during the suckling period but also contributing to antigen presentation and activation of the *naïve* immune system during and after weaning [22].

The immune system of suckling rats, 10 days old, is immature, whereas that of weaned rats, 21 days old, is comparable to that of an adult, suggesting a parallelism in timing between the activation of the immune system and gut maturation of the young at weaning [21, 28, 29]. In fact, there is a peak of interleukin-2 (IL-2) receptor expression on day 21 after birth [30] and experimental injection of IL-2 to suckling rat pups have been shown to induce precocious intestinal maturation, while the injection of the immunosupressive drug cyclosporine A, being an inhibitor of IL-2 production, reduces intestinal maturation [31, 32]. Moreover, IL-2 transcription was shown to be repressed by Blimp-1 in immune cells and to affect enterocyte gene expression in mice with defective adaptive immunity [33, 34]. Thus, the observations obtained in the present study, together with published data, suggest that the development of the SI epithelial barrier and the immune system of the gut not only show parallelism but also appear connected during postnatal period in rodents.

## 5. Conclusions

The switch from foetal- to adult-type SI epithelium during neonatal development with a decrease in FcRn expression in the proximal SI occurred in parallel to the disappearance of vacuolated enterocytes in the distal SI. This regional correlation of the developmental changes in the SI could be used in combination or separately for monitoring and estimating the stage of intestinal maturation in the neonatal rat. Even though Blimp-1 cannot be used as an ultimate marker for maturational monitoring, the changes in expression in the distal SI during maturational alterations suggest that Blimp-1 might be involved in the intestinal barrier development but also supports the connection with the immune system during the neonatal period in rats.

## Supporting Information

S1 Fig The specificity of the anti-Blimp-1 antibody was verified by neutralization with the Blimp-1 peptide control. Immunohistochemistry with and without blocking with the immunizing 14-aa Blimp-1 peptide (antibody: blocking peptide ratio; 1:5) in the distal part of the SI in suckling 14 days old and post-weaning 28 days old rats. In contrast to anti-Blimp-1 staining, the tissues incubated with the antibody pre-neutralized with the Blimp-1 peptide showed no staining. Negative control, incubation without Blimp-1 antibody is included as a secondary detection system control.
(TIF)

S2 Fig Replacement of vacuolated epithelium by non-vacuolated epithelium in the distal part of the SI during postnatal development in rats. H & E stained representative histological sections (200X) showing the structural changes in the proximal and distal parts of the SI during postnatal development in 7, 14, 21, 28 and 35 days (d) old rats.
(TIF)

S3 Fig Replacement of FcRn^pos^ epithelium by FcRn^neg^ epithelium in the SI during postnatal development in rats. Immunohistochemistry of FcRn (200X) in representative histological sections from the proximal and distal SI during postnatal development in 7, 14, 21, 28 and 35 days (d) old rats.
(TIF)

S4 Fig Changes in FcRn expression in the distal part of the SI of rats due to precociously induced maturation. Immunohistochemistry of FcRn (200X) in representative histological sections from the distal SI in 17 days old rats treated with PHA or protease at 14–16 days of age to induce precocious gut maturation, compared to control rats. Note the increased FcRn staining in the *lamina propria*, especially higher in the PHA treated group.
(TIF)

S5 Fig Correlation between FcRn expression and plasma IgG during postnatal development in rats. Changes of plasma IgG (mg/ml, mean ± SD) and appearance of adult-type cells (% FcRn^neg^ of total villi cells, mean ± SD) in the proximal SI during postnatal development in 7, 14, 21, 28 and 35 day old rats. Note the inverse correlation with a coefficient of r = -0.65.
(TIF)
