# Correction: Maturation of the Intestinal Epithelial Barrier in Neonatal Rats Coincides with Decreased FcRn Expression, Replacement of Vacuolated Enterocytes and Changed Blimp-1 Expression

**DOI:** 10.1371/journal.pone.0169724

**Published:** 2017-01-03

**Authors:** Ester Arévalo Sureda, Björn Weström, Stefan G. Pierzynowski, Olena Prykhodko

The images for Figs 6, 7 and 8 are incorrectly switched. The image that appears as Fig 6 should be Fig 7, the image that appears as Fig 7 should be Fig 8, and the image that appears as Fig 8 should be Fig 6. The figure captions appear in the correct order.

**Fig 6:**
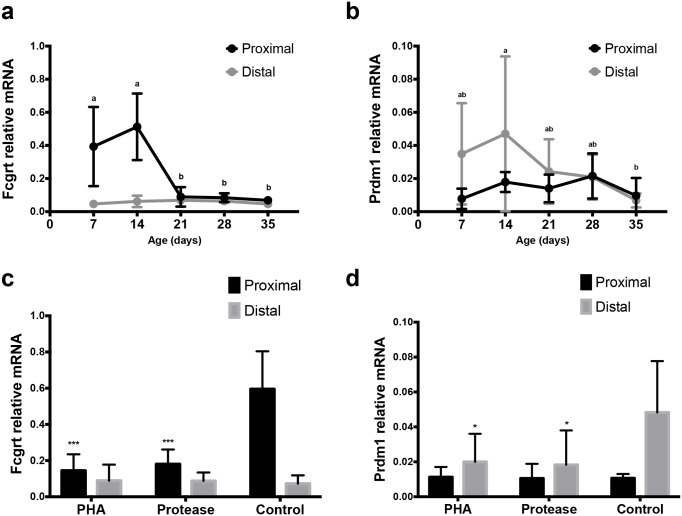
Expression of mRNA for Fcgrt (FcRn) and Prdm1 (Blimp-1) in the SI of developing rats. Relative mRNA levels of Fcgrt (a, c) and Prdm1 (b, d) in the proximal and distal SI in 7, 14, 21, 28 and 35 days old rats (n = 4–6) during postnatal development (a, b) and in 17 days old rats treated with PHA or protease at 14–16 days of age (n = 3–4) to induce precocious gut maturation, compared to control rats (c, d). (mean ± SD, a–b and * p < 0.05; *** p < 0.001).

**Fig 7:**
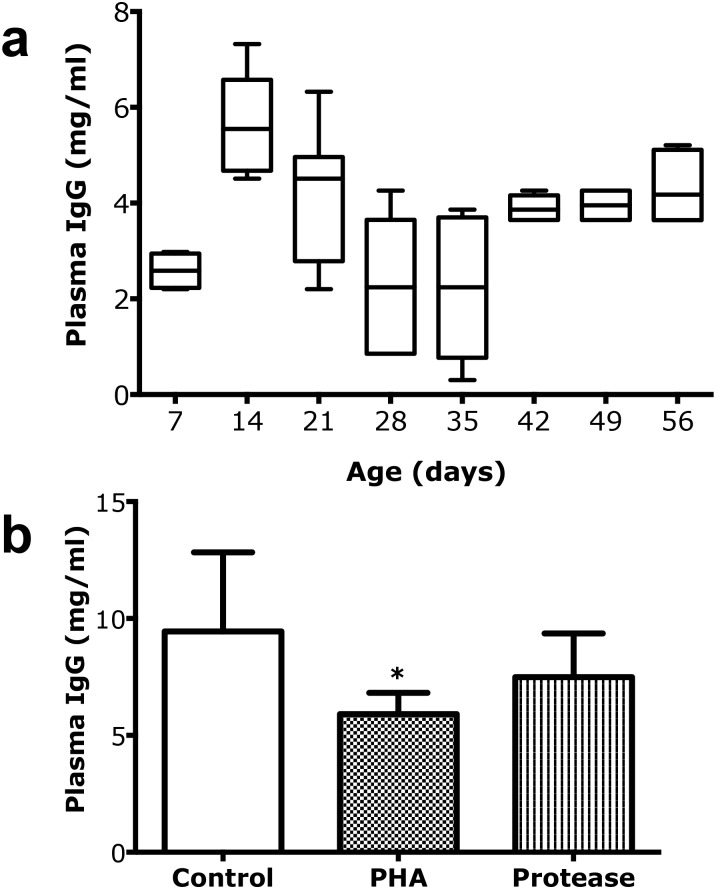
Plasma IgG as an accumulative marker for intestinal macromolecular permeability. (a) Changes in plasma IgG levels (mg/ml) during postnatal development in suckling rats, 7, 14, and 21 days old and post-weaning rats, 28, 35, 42, 49 and 56 days old (mean ± SD, 10–90% percentile, (boxes), n = 6–10). (b) Decreased plasma IgG levels (mean ± SD, n = 6) in 17 days old rats treated with PHA (* p < 0.05) or protease (non-significant) during 3 days to induce precocious maturation, compared to control rats.

**Fig 8:**
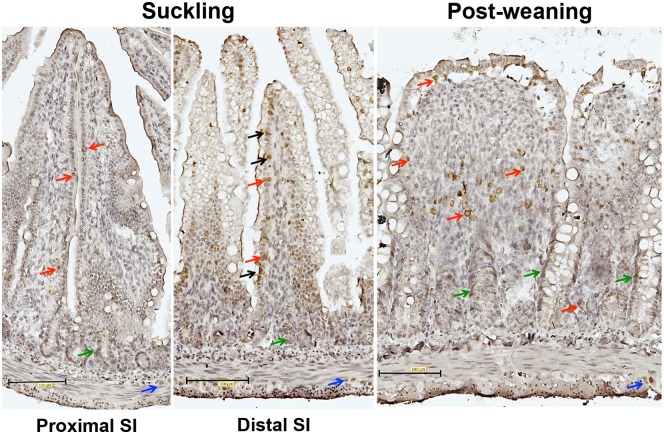
Expression of Blimp-1 in the small intestine during suckling and post-weaning periods. Immunohistochemical staining of Blimp-1 in representative histological sections (magnification 200x) from the proximal and distal SI in suckling (14d) and post-weaning (28d) rats. Note: Nuclear immunorreactivity of Blimp-1 antibodies in epithelial cells indicated by black arrows; intraepithelial and lamina propria cells–red arrows; crypt cells–green arrows and cells in muscle layer are indicated by blue arrows.
