# Supplementary figures and images for: Maturation of the Intestinal Epithelial Barrier in Neonatal Rats Coincides with Decreased FcRn Expression, Replacement of Vacuolated Enterocytes and Changed Blimp-1 Expression
Source: PLoS One. 2016 Oct 13;11(10):e0164775. doi: 10.1371/journal.pone.0164775 (PMC5063338; doi:10.1371/journal.pone.0164775)

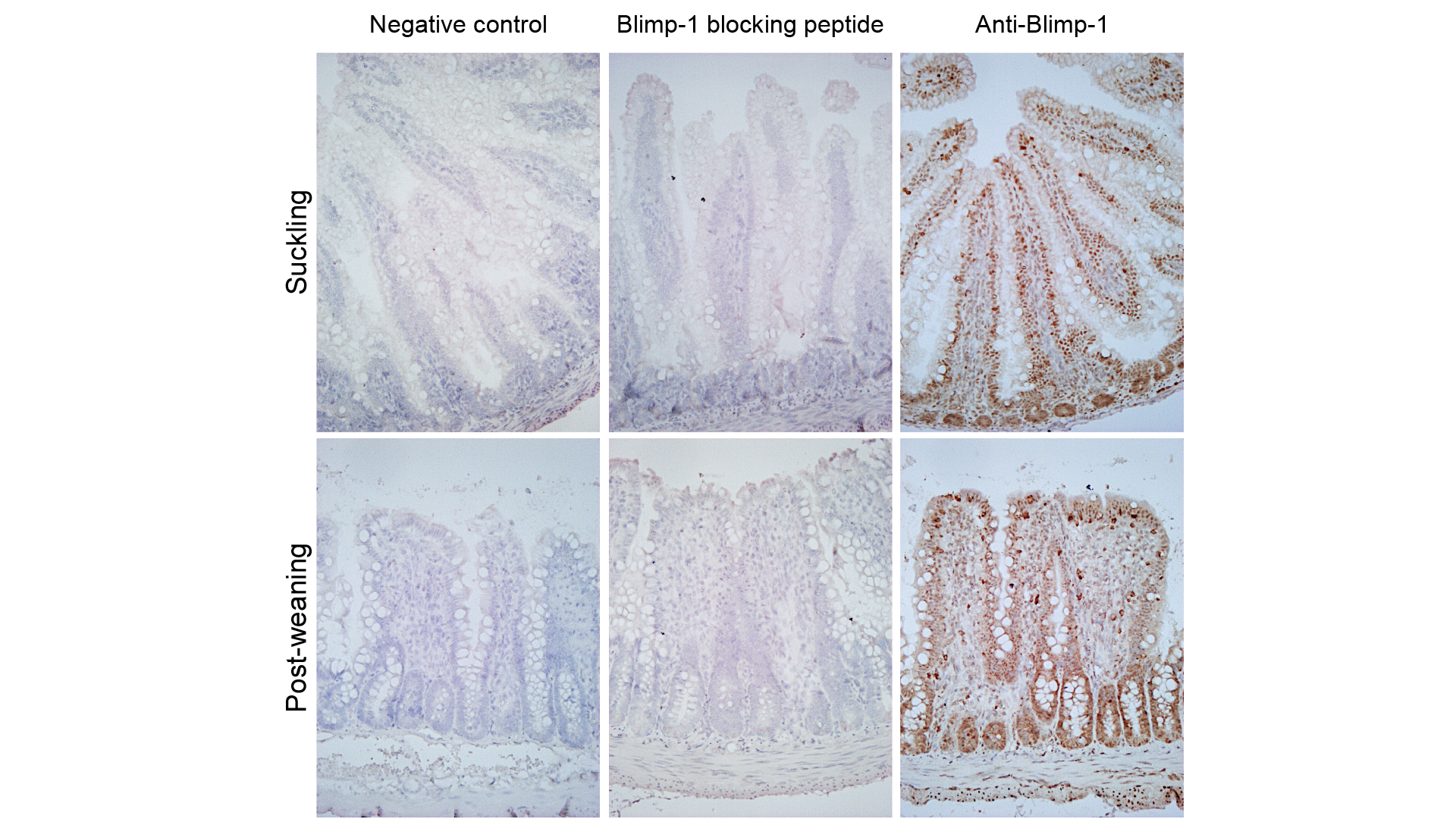

Supplement: S1 Fig — Immunohistochemistry with and without blocking with the immunizing 14-aa Blimp-1 peptide (antibody: blocking peptide ratio; 1:5) in the distal part of the SI in suckling 14 days old and post-weaning 28 days old rats. In contrast to anti-Blimp-1 staining, the tissues incubated with the antibody pre-neutralized with the Blimp-1 peptide showed no staining. Negative control, incubation without Blimp-1 antibody is included as a secondary detection system control. (TIF) [file pone.0164775.s001.tif]

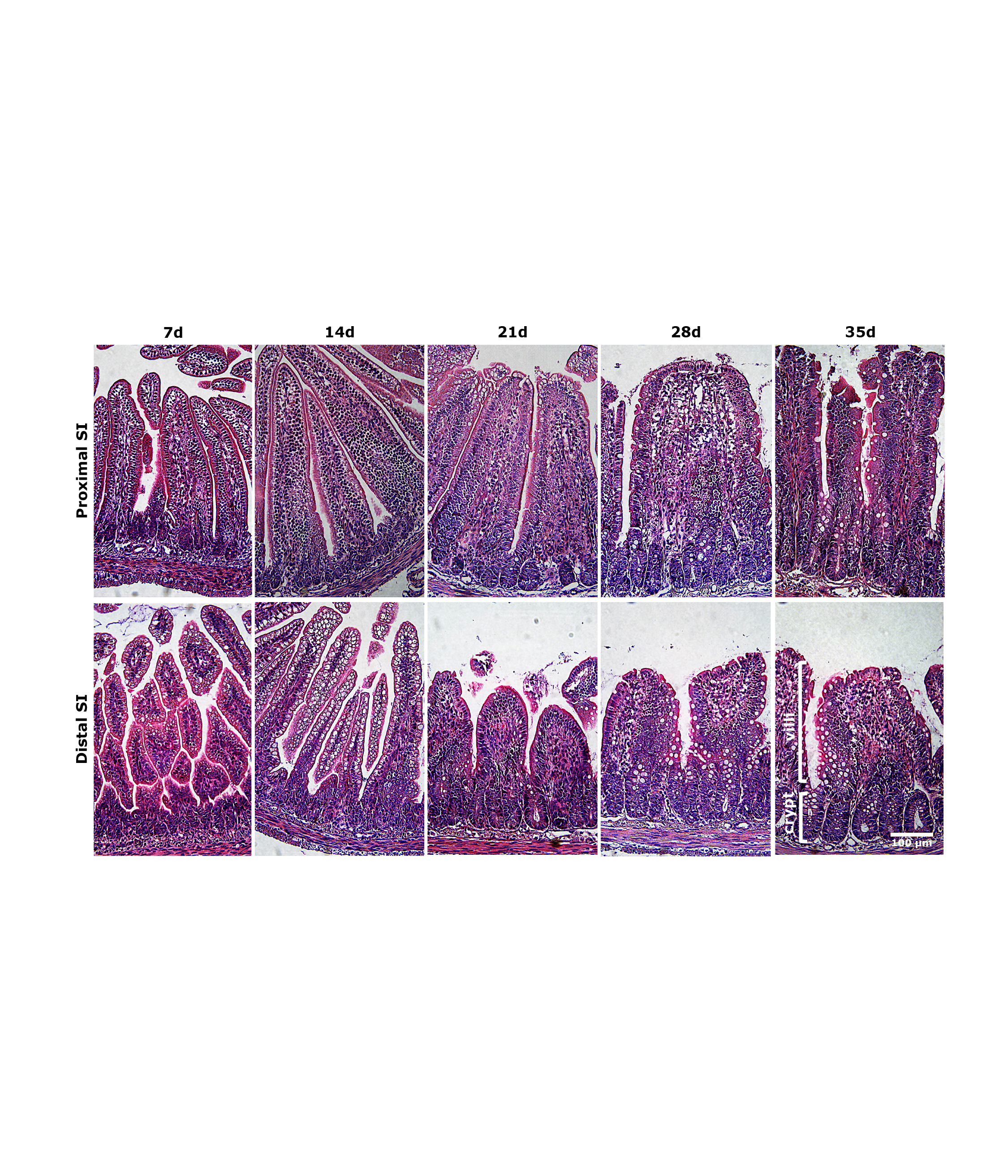

Supplement: S2 Fig — H & E stained representative histological sections (200X) showing the structural changes in the proximal and distal parts of the SI during postnatal development in 7, 14, 21, 28 and 35 days (d) old rats. (TIF) [file pone.0164775.s002.tif]

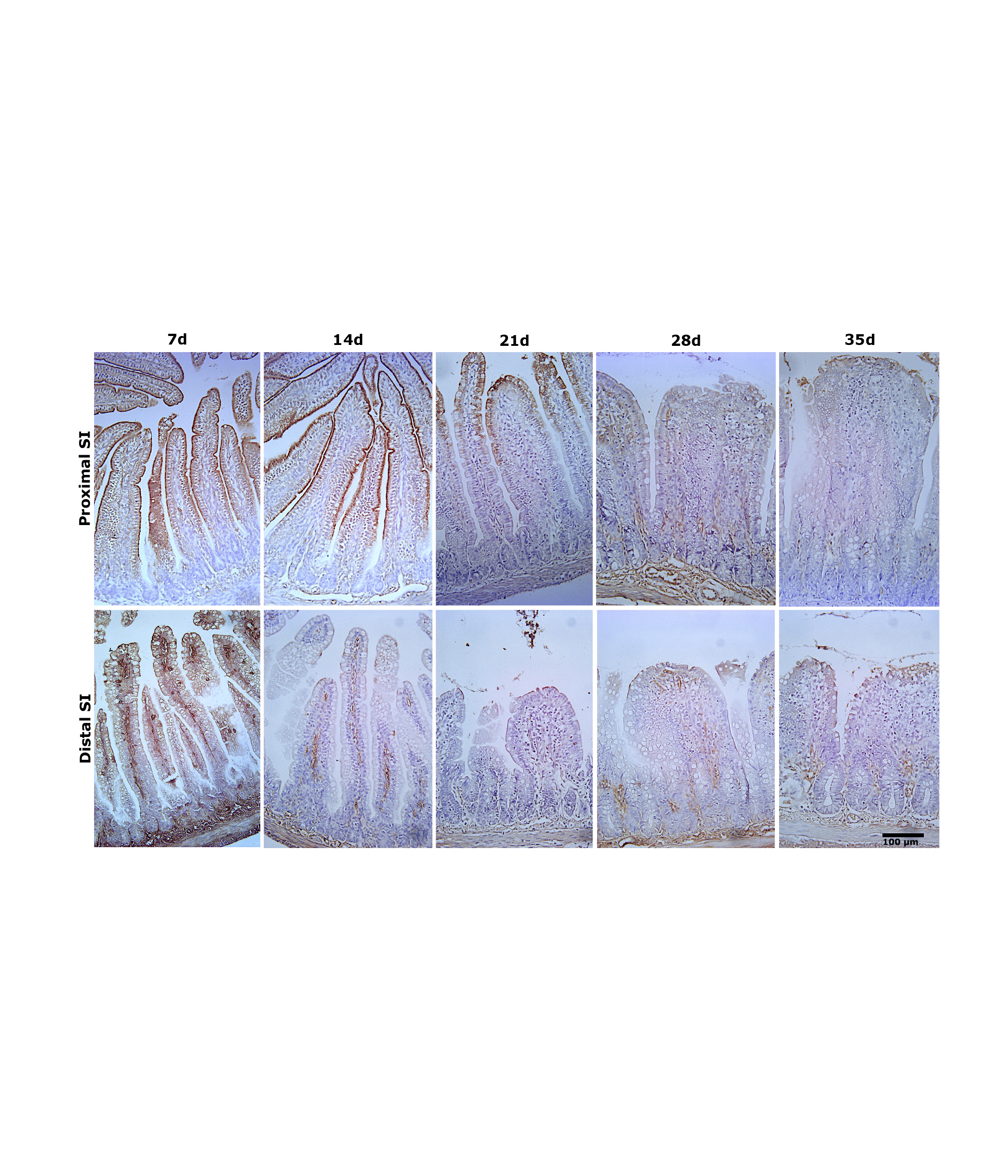

Supplement: S3 Fig — Immunohistochemistry of FcRn (200X) in representative histological sections from the proximal and distal SI during postnatal development in 7, 14, 21, 28 and 35 days (d) old rats. (TIF) [file pone.0164775.s003.tif]

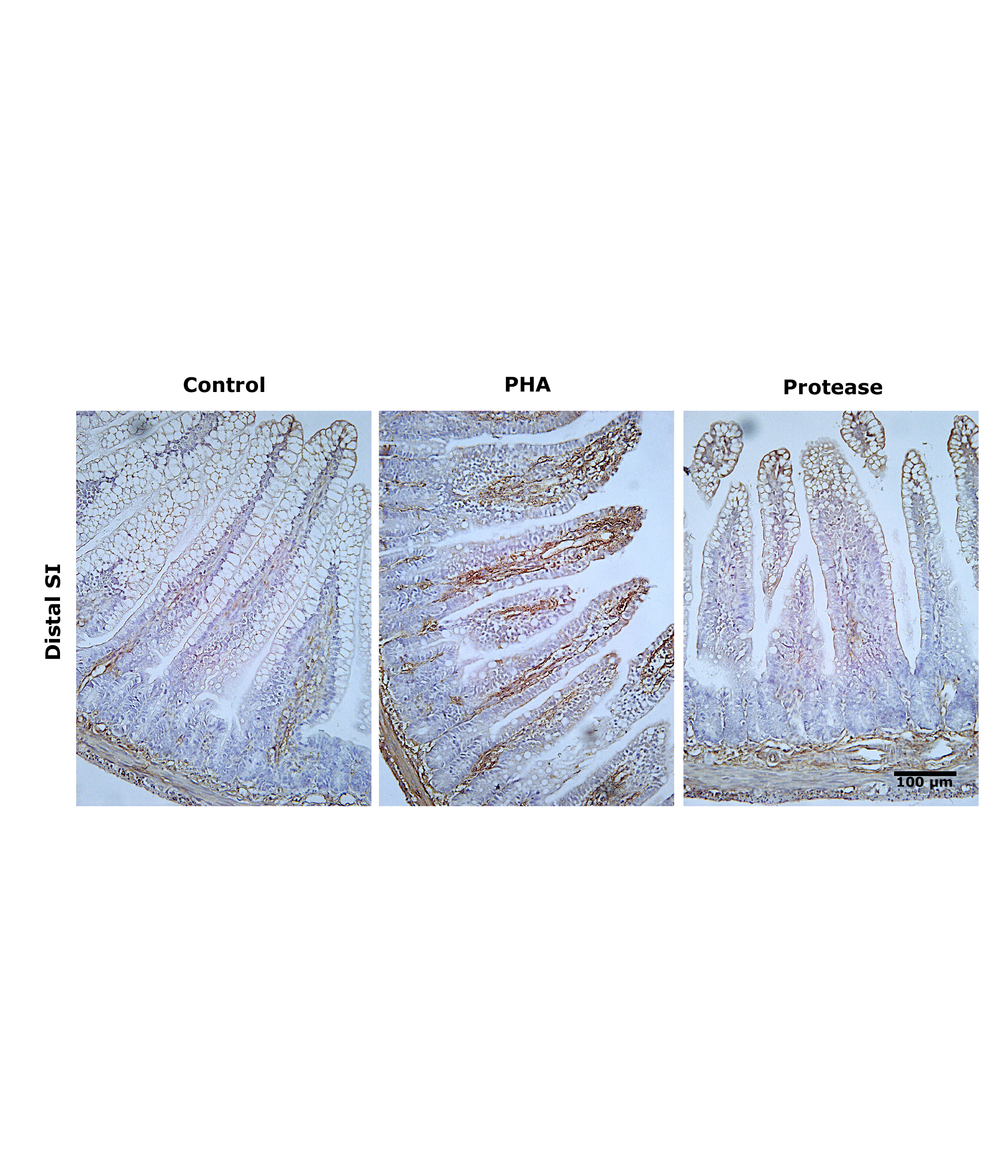

Supplement: S4 Fig — Immunohistochemistry of FcRn (200X) in representative histological sections from the distal SI in 17 days old rats treated with PHA or protease at 14–16 days of age to induce precocious gut maturation, compared to control rats. Note the increased FcRn staining in the lamina propria, especially higher in the PHA treated group. (TIF) [file pone.0164775.s004.tif]

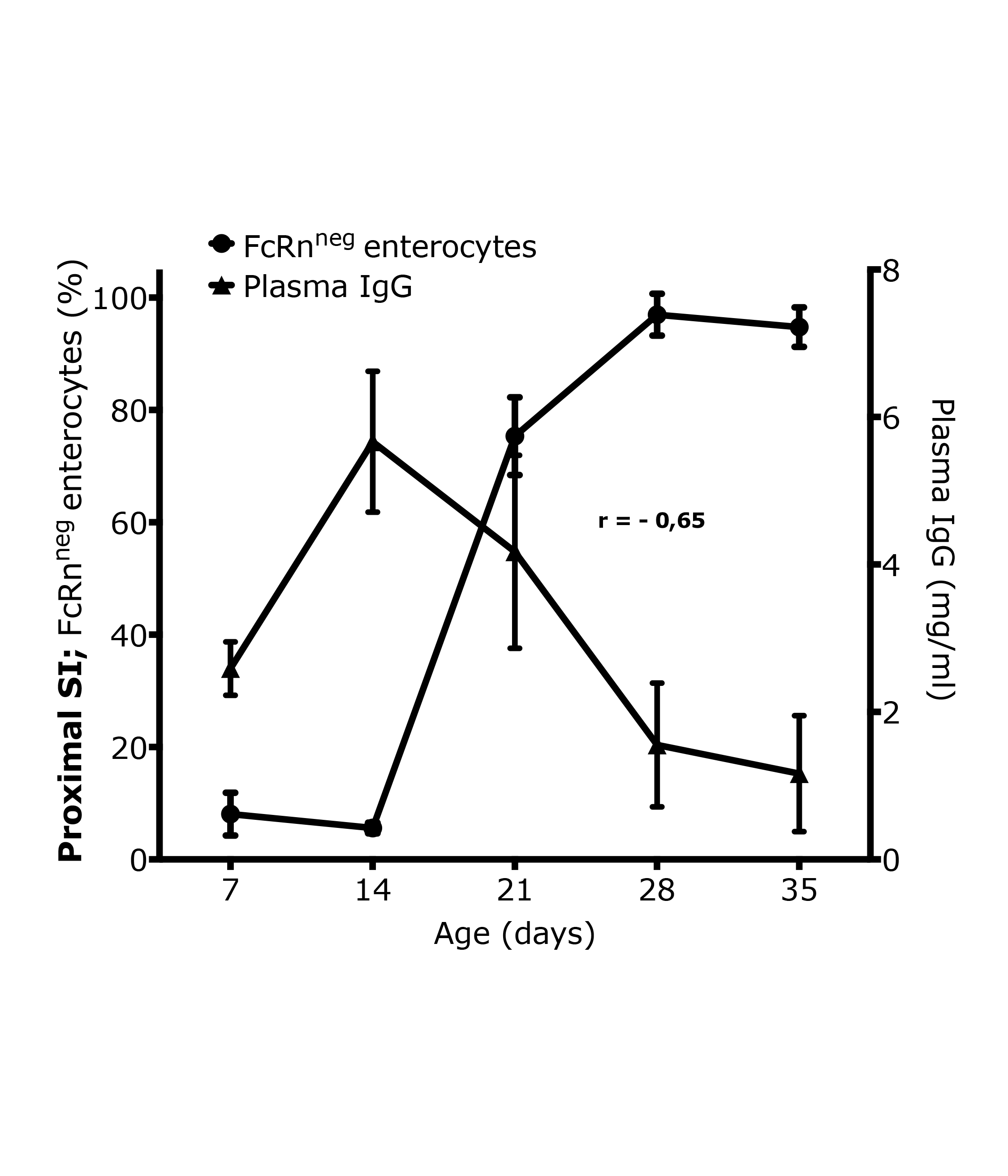

Supplement: S5 Fig — Changes of plasma IgG (mg/ml, mean ± SD) and appearance of adult-type cells (% FcRnneg of total villi cells, mean ± SD) in the proximal SI during postnatal development in 7, 14, 21, 28 and 35 day old rats. Note the inverse correlation with a coefficient of r = -0.65. (TIF) [file pone.0164775.s005.tif]
